# Key Associations Found in the Struggle With Sleep in Lung Transplant Recipients

**DOI:** 10.1177/15269248241289149

**Published:** 2024-10-15

**Authors:** Jane Simanovski, Jody Ralph, Sherry Morrell

**Affiliations:** 1 Faculty of Nursing, 8637 University of Windsor, Windsor, Canada; 2 Transplant Institute, 2971 Henry Ford Hospital, Detroit, MI, USA

**Keywords:** sleep quality, lung transplantation, anxiety, Hospital Anxiety and Depression Scale, Pittsburgh Sleep Quality Index, research, quantitative methods, regression, systems, health

## Abstract

**Introduction:**

Gaps exist in the understanding of the etiology of poor sleep quality after lung transplantation. Research Question: What factors are associated with poor sleep quality in lung transplant recipients?

**Design:**

A quantitative, single-site, cross-sectional study used an anonymous survey based on 3 scales. The Pittsburgh Sleep Quality Index scale with scores dichotomized to poor versus good sleepers based on the cutoff score > 8. The Hospital Anxiety and Depression Scale evaluated symptoms of anxiety and depression, and the Short Form-12 measured health-related quality of life using the mental and physical component scores. Additional self-reported data included demographic and transplant-related variables.

**Results:**

The response rate was 38.4% (61/158), and 52.5% of the sample (32/61) evidenced a Pittsburgh Sleep Quality Index score > 8, suggestive of poor sleep quality. Bivariate analyses demonstrated that poor sleep was significantly related to symptoms of depression (*P* < .01), anxiety (*P* < .01), stressors of hospitalization (*P* < .05), and treatment of acute rejection (*P* < .05). Multivariate analysis demonstrated that anxiety was significantly associated with poor sleep (odds ratio = 1.34, *P* < .05).

**Conclusion:**

Poor subjective sleep quality remains prevalent in lung transplant recipients. Individuals with anxiety symptoms were at a greater risk for poor sleep. Guidance for strategies to improve sleep quality requires further in-depth exploration before implementation of interventions.

## Introduction/Background

Sleep has been recognized for its impact on many aspects of health and wellness.^1,2^ The circulatory, respiratory, musculoskeletal, and central nervous systems are repaired during sleep.^1^ Poor sleep is associated with adverse health outcomes such as obesity, diabetes, cardiovascular disease, impaired immunity, neurocognitive dysfunction, depression, anxiety, poor quality of life, and increased risk of death.^2–4^ Subjective sleep quality is an individual's perception of sleep that captures a variety of quantitative and subjective components of sleep, such as difficulty falling and staying asleep, sleep duration, nighttime awakening, whether sleep is perceived as restful, and its effect on daytime functioning.^5^

Many adversities can arise post-lung transplantation. These include extended hospital stays, readmissions, immunosuppressive medications, and complications such as primary graft dysfunction, acute cellular and antibody medicated rejections, infections, diabetes, cardiovascular disease, and gastrointestinal complications^6,7^ contributing to morbidities and poor quality of life. Heightened emphasis is placed on identifying and optimizing modifiable risk factors to improve survival and quality of life after transplant.^8^ Diminished sleep quality and sleep disturbances can impact quality of life and may be associated with emotional and physical illness.^5^ Significant gaps exist in the understanding of sleep after lung transplantation. A recent scoping^9^ review identified 12 sources that addressed sleep quality or its components in lung transplant recipients. The results of this scoping review suggested that lung transplant patients experienced poor sleep and confirmed that this area of research needs further study.^9^ If sleep is one of the modifiable risk factors for improving survival and quality of life after transplant, suboptimal sleep quality poses an understudied and potentially essential element to be addressed in post-transplant care.^8,9^

Considering the significance of sleep on health and well-being, this study was initiated to determine which patient factors had a relationship with poor sleep in lung transplant recipients.

## Design/Methods

### Design

This quantitative survey research was a single-site, cross-sectional study involving lung transplant recipients followed at a transplant center in the Midwest region of the United States. The study received Institutional Review Board (IRB) (# 15510) approval at the transplant center and clearance from the Research Ethics Board (# 22-055) at the University. Given the anonymous nature of the survey that subjects were asked to complete, this research qualified for exempt status and received a waiver for the documentation of the informed consent. Each participant was still provided with the required consent information with the survey, but the study team was not required to obtain the participant's signature on the informed consent document; completion of the survey demonstrated participant consent.

### Setting

Lung transplant recipients at an established midwestern transplant center were invited to participate in an anonymous survey. Although potential subjects were given information about the study during their clinical encounters or through a mailed flyer, they could complete the survey on their personal devices, such as computers or smartphones, which ensured their privacy.

### Population

The target population was adults who received a lung transplant where 453 transplants have been performed since the inception of this lung transplant program in 1994. The center's data indicates that the majority of the lung transplant recipients were White (356/453); between the ages of 50 to 64 (251/453); and males (276/177).^10^ The program's 1-year survival rate is 93% with 3-year survival rate 79.41% consistent with national trends according to the data from the United States Organ Procurement and Transplantation Network.

### Sampling

**Figure 1.**
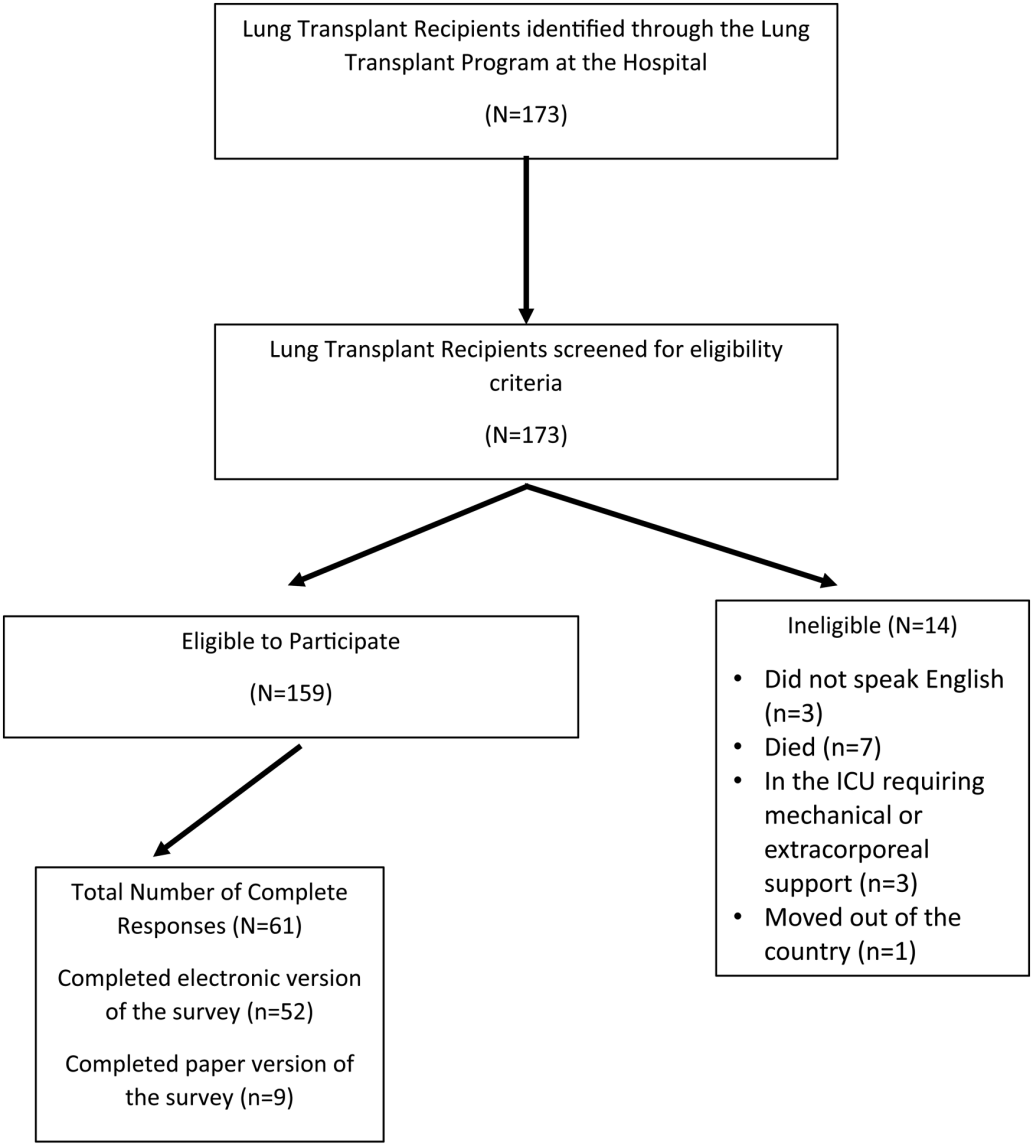
Eligibility to participate schema.

The following inclusion criteria were used for eligibility: lung transplant recipient regardless of year of transplantation; followed at the lung transplant program; adult  ≥ 18 years; able to understand, read, and write in English; and able to provide informed consent. Both outpatients and inpatients were eligible to participate, unless critically ill in the intensive care unit requiring life-sustaining invasive therapies, such as mechanical ventilation, extracorporeal life support, and vasoactive medications. Subjects were recruited between May 2022 and September 2022. Prospective subjects were approached by the principal investigator either during their scheduled clinic visits at the transplant clinic or by a flyer mailed to their home address. Every effort was made to approach patients in person to maximize face-to-face contact using the standardized recruitment script. Mass communication was sent out electronically via the patient communication portal in EPIC (Epic Systems Corporation, Verona, WI) with an information letter, link to the survey, and the recruitment flyer about the study containing the Quick Response (QR) code. **Figure 1** illustrates the eligibility for study participation.

### Data Collection

#### Measures

*Sample Characteristics.* Sample characteristics were captured self-report data and included general demographic questions (eg, age, sex at birth, marital status, race) and inquiries such as type of transplant, presence of co-morbidities, changes to immunosuppressive medications, use of non-pharmacological sleep aids, exercise, and situational stressors due to their possible relationship with sleep quality.

*Sleep quality: Pittsburgh Sleep Quality Index (PSQI).* Subjective sleep quality was captured from the PSQI global score. The PSQI is the most widely employed subjective measure of sleep dysfunction and the only standardized clinical instrument that covers a range of indicators relevant to sleep quality.^3^ The PSQI is a 19-item self-report questionnaire assessing global sleep quality score and 7 component scores: sleep quality, sleep latency, sleep duration, habitual sleep efficiency (the ratio of sleep duration by time spent in bed), sleep disturbance (eg, using the washroom), use of sleeping medications, and daytime dysfunction.^5^ Although not tested in this population, the psychometric properties, have been tested in similar populations, including bone marrow and renal transplant recipients, supporting PSQI's internal consistency, reliability, and construct validity.^11^ The PSQI was internally consistent with Cronbach's alpha coefficients ranging from 0.80 to 0.83 in various populations, including solid organ transplant recipients.^5,11^ A global PSQI score of 5 or higher provides a sensitive and specific measure of poor sleep quality.^5^ Carpenter and Andrykowski^11^ postulated that mean PSQI scores were above 8 (rather than 5) in bone marrow and renal transplant recipients with sleep problems, suggesting that 8 might be a more appropriate cutoff score to indicate poor sleep quality in individuals with chronic health conditions. Hence, to determine the proportion of lung transplant recipients with good versus poor sleep, PSQI global score was dichotomized using the established threshold of less than or equal to 8 (0-8) to classify good sleepers versus PSQI score greater than 8 (9-21) as a marker of poor sleep quality. Permission to use the PSQI in this study was granted by Daniel J. Buysse, M.D., Professor of Psychiatry and Clinical and Translational Science, University of Pittsburgh School of Medicine

*Health-related quality of life measure: Short Form-12 (SF-12).* Health-related quality of life (HRQoL) in transplantation is a multi-dimensional concept without an agreed-upon operational definition.^12,13^ Self-reported HRQoL was measured using Short Form-12 (SF-12)—a multipurpose generic measure of health and well-being with its Physical and Mental Component Summary scores (PCS and MCS).^14^ It is an abbreviated version of the Short Form-36 and was developed to provide an alternative to the longer form with ease of administration and the same level of reliability.^14^ The SF-12 is comprised of 12 items that compose 8 subscales that make up the measure. The 8 subscales include Role Physical, Bodily Pain, General Health, Vitality, Social Functioning, Role Emotional, and Mental Health. They are then weighted and summarized into 2 component scores: The PCS and the MCS. These are norm-based scores for the general US population, with a mean of 50 and a standard deviation (SD) of 10. The scores range from 0 to 100, with higher scores indicating better HRQoL. In the development of the SF-12 instrument, Ware et al^14^ reported that tests of validity previously published for the Short Form-36 scales and summary measures were replicated for the SF-12 instrument. Upon obtaining permission to use the SF-12 questionnaire from Quality Metrics (Johnston, RI), PROCoRE scoring software was provided.

*Depression and anxiety: Hospital Anxiety and Depression Scale (HADS).* Symptoms of anxiety and depression were measured using the HADS.^15^ The scale contains 14 items on 2 subscales: anxiety (7 items) and depression (7 items). Based on the original HADS publication, a score of above 8 on either subscale (anxiety or depression) indicates possible or borderline symptoms, while a score of above 10 indicates probable clinical symptoms.^15^ Reliability analysis revealed an acceptable internal consistency level for the HADS anxiety (Cronbach's alpha = 0.82) and depression (Cronbach's alpha = 0.73) subscales in this study. Permission to use the HADS from MapiTrust (Lepardstown, Dublin, IE) was secured.

#### Data Analysis

All data were exported and analyzed using SPSS V28.0 (IBM, Armonk, NY). The data analysis was conducted in 3 phases. In the first step of the analysis, continuous variables (interval/ratio level) were presented using descriptive statistics such as means (*M*), standard deviation (SD), and minimum/maximum (MIN/MAX) values. Categorical variables (nominal/ratio level) were described with frequencies and percentages. The second step of data analysis was bivariate analysis. Bivariate analysis involved identifying which explanatory variables were related to each dependent variable at a statistically significant level (*P *< .05). The relationship between 2 categorical variables was examined via chi-square analysis. Pearson's r zero-correlation was used to examine the relationship between 2 continuous variables. An independent samples t-test examined the relationship between a dichotomous and continuous variable. Each explanatory variable that was found to be significantly related to each respective dependent variable was included in the third phase of data analysis for that dependent variable, which was multivariate analysis. A binary logistic regression model involved modeling the dependent variable Poor Sleep Quality (Yes/No*)* as a function of the explanatory variables significantly related to that dependent variable in bivariate analysis. The G*power software indicated that a medium effect size effect (O = 3.00) would be detected within a logistic regression model with power set at .80 and alpha set at .05, using a sample size of 53 study subjects. Thus, the current sample of 61 study subjects provided sufficient statistical power for the analysis. Within the final inferential analysis presented, the parametric test assumptions of normality, linearity, homoscedasticity, multicollinearity, and no undue influence of outlier scores were examined and revealed no significant problems.

#### Procedure

Study data were collected and managed using the REDCap electronic data capture tool (Vanderbilt University, Nashville, TN) hosted at the transplant site. Surveys were anonymous and took approximately 20 min to complete. The paper version of the survey was also available at subjects’ request whereby they were provided with a paper version of the same survey and a pre-paid addressed envelope to mail back to the principal investigator. Once the paper version of the survey was received via mail, the data was manually added into the REDCap. No identifying data were collected on the electronic or paper version. All entries were double-checked for accuracy and verified by the second author. Upon finishing the survey, subjects were informed they were eligible for a mailed Target Gift Card valued at $15 as a token of appreciation for participating in the study.

## Results

### Sample Characteristics

Of the 173 patients identified as lung transplant recipients followed at the transplant center, 159 met the inclusion criteria to be eligible for the study. The response rate to the survey was 38.4% (N = 61).

Characteristics of the sample and questionnaire responses are presented in **Tables 1** and **2**. Subjects’ ages ranged from 30 to 77 years (*M *= 61.46). Almost half of the study subjects (N = 27, 44.3%) were 1 to 3 years since their lung transplant, and most transplant types were bilateral (N = 57, 93.4%). The sample was predominantly male (N = 41, 67.2%), married (N = 42, 68.9%), and White in race (N = 47, 77.0%).

**Table 1.** Participant Characteristics.

Variable	*N* (%)	Mean (SD)
**Study participant age**		61.46 (10.16)
**BMI** (N = 59, 2 missing)		27.61 (5.45)
**Sex assigned at birth**		
Male	41 (67.2)	
**Marital status**		
Married	42 (68.9)	
Single	11 (18)	
Divorced	4 (6.6)	
Living with a partner	4 (6.6)	
**Race**		
White	47 (77.0)	
Black	7 (11.5)	
Hispanic ethnicity	2 (3.3)	
Other race or ethnicities	5 (8.2)	
**Years since transplant**		
Less than 1 year	13 (21.7)	
1-3 years	27 (45.0)	
4-5 years	6 (10.0)	
6 or more	14 (23.3)	
**Transplant type**		
Double	57 (93.4)	
**Current oxygen use**		
No	57 (93.4)	
**Co-morbid condition**		
Diabetes	23 (37.7)	
Cardiovascular disease	22 (36.1)	
Acid reflex	30 (49.2)	
Diagnosed sleep disorder	13 (21.3)	
**Stressor (responded yes)**		
Hospitalization in the last 30 days	14 (23.0)	
Treatment of acute rejection in the last 30 days	4 (6.6)	
Illness of a family member	10 (16.4)	
Additional self-reported stressors	12 (19.7)	
**Immunosuppression changes in the last 30 days**		
Yes	17 (27.8)	
**Sleep aids used in the Past 30 days**		
Use of alcohol, Yes	0 (0)	
Use of cannabinoids, No	58 (95.1)	
Use of herbs, No	58 (95.1)	
**Poor sleep quality (based on the results of PSQI scores)**		
Yes (PSQI scores 9 thru 21)	32 (52.5)	
No (PSQI scores 0 thru 8)	29 (47.5)	

Abbreviations: BMI, body mass index; PSQI, Pittsburgh Sleep Quality Index.

**Table 2.** Response Scores to Health-related Quality of Life and Anxiety/Depression Questionnaires (N = 61).

Variable	M (SD)	Minimum/ Maximum	Skew (SE)	Kurtosis (SE)
MCS scores	52.65 (9.85)	23.57-66.28	−1.08 (.31)	.58 (.60)
PCS scores	42.37 (8.78)	25.11-61.06	.02 (.31)	−.87 (.60)
HADS depression scores	3.02 (2.44)	0.00-9.00	.81 (.31)	−.08 (.60)
HADS anxiety scores	5.13 (3.40)	0.00-13.00	.44 (.31)	−.36 (.60)

Abbreviations: HADS, hospital anxiety and depression scale; MCS, mental component scores; PCS, physical component scores.

More than half of the study subjects (N = 32, 52.5%) evidenced poor sleep quality based on the PSQI cutoff of greater than 8. The average SF-12 Mental Component Score was 52.65 (SD = 9.85, MIN/MAX = 23.57-66.28), SF-12 Physical Component Score was 42.37 (SD = 8.78, MIN/MAX = 25.11-61.06), HADS depression score was 3.02 (SD = 2.44, MIN/MAX = 0.00-9.00), and HADS anxiety score was 5.13 (SD = 3.40, MIN/MAX = 0.00-13.00). The distributions of all the independent and dependent variable scores were approximately normal, as the skewness and kurtosis were not approximately 3 times the respective standard error of each value.

### Relationships With Poor Sleep Quality

**Tables 3** and **4** present bivariate analyses: a chi-square analysis of Poor Sleep Quality (Yes/No) by categorical explanatory variables and an independent samples t-test analysis of Poor Sleep Quality (Yes/No) by continuous explanatory variables. Poor Sleep Quality was significantly related to the stressors of: (*a*) hospitalization, X*²*(1) = 4.97, *P *< .05, with a higher percentage of poor sleep among the study subjects who reported hospitalization (Yes) (78.6%, = 11) relative to those who reported no hospitalization (No) (44.7%, n = 21); and (*b*) treatment of acute rejection, X*²*(1) = 3.88, *P *< .05, with a higher percentage of poor sleep among the study subjects who reported treatment of acute rejection (n = 4) (*Yes*) (100.0%, n = 4) relative to those who reported no treatment of acute rejection (No) (49.1%, n = 28). Data indicated Poor Sleep Quality (Yes/No) was significantly related to HADS depression scores, *t*(55.42) = 2.88, *P *< .01, with higher mean HADS depression scores among study subjects with poor sleep (Yes) (*M *= 3.81, SD = 2.66) relative to those without (No) (*M *= 2.14, SD = 1.85). Poor Sleep Quality was also significantly related to HADS anxiety scores, *t*(59) = 4.25, *P *< .001, with higher mean HADS anxiety scores among study subjects with poor sleep (Yes) (*M *= 6.69, SD = 3.43) relative to those without self-reported sleep difficulties (No) (*M *= 3.41, SD = 2.44). Lastly, poor sleep quality was significantly related to MCS of the SF-12, *t*(48.75) = -2.92, *P *< .01, with lower mean MCS among study subjects with poor sleep (Yes) (*M *= 49.43, SD = 11.42) relative to those without poor sleep quality (No) (*M *= 56.21, SD = 6.2).

**Table 3.** Bivariate Analyses. Chi-Square Analysis of Poor Sleep Quality (No/Yes by Categorical Explanatory Variables (N = 61).

	Poor sleep quality			
Variable	No	Yes	X² (df)	*P *
**Years since transplant **			1.76 (2)	.42
Less than 1 year	5 (38.5)	8 (61.5)		
1-3 years	12 (44.4)	15 (55.6)		
4 years or more	12 (60.0)	8 (40.0)		
**Transplant type **			.01 (1)	.92
Double	27 (47.4)	30 (52.6)		
**Assigned sex at birth, male **	22 (53.7)	19 (46.3)	1.88 (1)	.17
**Marital status **			5.78 (3)	.12
Single	4 (36.4)	7 (63.6)		
Married	22 (52.4)	20 (47.6)		
Divorced	3 (75.0)	1 (25.0)		
Living with a partner	0 (0.0)	4 (100.0)		
**Race, White**	22 (46.8)	25 (53.2)	.04 (1)	.83
**Current oxygen use, No**	27 (47.4)	30 (52.6)	.23 (1)	.64
**Presence of co-morbidity**				
Diabetes, No	19 (50.0)	19 (50.0)	.24 (1)	.62
Cardiovascular, No	22 (56.4)	17 (43.6)	3.41 (1)	.07
Acid reflex, No	16 (51.6)	15 (48.4)	.42 (1)	.52
Sleep disorder, No	22 (45.8)	26 (54.2)	.26 (1)	.61
Stressor: Hospitalization, No	26 (55.3)	21 (44.7)	4.97 (1)	.03
**Presence of stressors in the last 30 days**				
Treatment of acute rejection, No	29 (50.9	28 (49.1)	3.88 (1)	.05
Illness of family member, No	26 (51.0)	25 (49.0	1.48 (1)	.22
Other unspecified, No	24 (49.0)	25 (51.0)	.65 (1)	.21
**Use of sleep aids in the last 30 days**				
Alcohol, No	61	52.65 (9.85)	NA (NA)	NA
Cannabinoids, No	29 (50.0)	29 (50.0)	2.86 (1)	.09
Herbs, No	29 (50.0)	29 (50.0)	2.86 (1)	.09
**Medicine changes, No**	23 (54.8)	19 (45.2)	3.12 (1)	.08
**Attended an exercise program in the last 30 days **	14 (46.7)	16 (53.3)	.0 (1)	1.00

**Table 4.** Bivariate Analyses. Independent Samples T-Test Analysis of Poor Sleep Quality (No/Yes) by Continuous Explanatory Variables (N = 61).

	Poor sleep quality			
	No (N = 29)	Yes (N = 32)		
Variable	M (SD)	M (SD)	t (df)	*P*
Participant age	63.82 (7.46)	59.31 (11.82)	−1.76 (59)	.08
BMI	26.82 (5.80)	28.38 (5.06)	1.10 (57)	.28
HADS depression scores	2.14 (1.85)	3.81 (2.66)	2.88 (55.42)	.006*
HADS anxiety scores	3.41 (2.44)	6.69 (3.43)	4.25 (59)	.001**
MCS scores	56.21 (6.20)	49.43 (11.42)	−2.92 (48.75)	.005*
PCS scores	44.00 (9.30)	40.90 (8.15)	−1.39 (59)	.17

Abbreviations: BMI, body mass index; X², chi-square; df, degrees of freedom; HADS, hospital anxiety and depression scale; MCS, mental component scores; PCS, physical component scores. The variable treatment of acute rejection was not included in the multivariate model because one of the cells in the 2 × 2 distribution contained 0 cases.

* *P *< .01.

** *P *< .001.

**Table 5** presents a binary logistic regression analysis examining Poor Sleep Quality (Yes/No). Analysis indicated that the overall model was statistically significant, X*²*(4) = 20.69, *P *< .001, and 72.1% of the cases were categorized correctly. Regarding the individual predictors, higher HADS anxiety scores were significantly associated with a greater likelihood of reports of poor sleep quality (Yes), B = 0.29, SE = 0.13, Wald X*² *= 5.29, odds ratio = 1.34, 95% CI = 1.04 to 1.72, *P *< .05.

**Table 5.** Multivariate Analysis: Binary Logistic Regression Analysis Examining Poor Sleep Quality—Yes/No (N = 61).

Variable	B (SE)	Wald X²	OR (95% CI)	*P*
Stressor: Hospitalization	1.46 (.81)	3.28	4.30 (.89-20.87)	.07
HADS depression scores	−.02 (.20)	.02	.98 (.66-1.44)	.90
HADS anxiety scores	.29 (.13)	5.29	.98 (.66-1.44)	.02*
MCS scores	−.05 (.05	0.85	.96 (.87-1.05)	.36

Note. For Model: *X²*(4) = 20.69, *P *< .001. 72.1% of the cases were categorized correctly.

Abbreviations: B, beta coefficient X; HADS, hospital anxiety and depression scale; MCS, mental component scores.

* *P *< .01.

## Discussion

Consistent with the limited literature, these results suggested that over half of the study subjects (N = 32, 52.5%) evidenced poor sleep quality based on the PSQI cutoff greater than 8. This is in line with other studies using the PSQI to measure subjective sleep quality, where previous authors reported that poor sleep was present in 32%-81% of lung transplant recipients.^16–19^ Two articles used a PSQI cutoff greater than 5^18,19^; Fatigati et al^16^ and Reilly-Spong et al^17^ used a cutoff score greater than 8.

A significant association was found between stressors of hospitalization and treatment of acute rejection and poor sleep quality. When asked about hospitalization, the question was phrased to ask whether individuals experienced hospitalization within the preceding 30 days. Therefore, it was challenging to ascertain which individuals were actively under inpatient hospital status versus recently discharged from the hospital. Some subjects might have filled out this survey while admitted to the inpatient service, as hospitalization alone did not exclude individuals willing to participate. Similarly, the authors of a multicenter study of 2005 patients reported that hospitalized patients experienced shorter durations of sleep, woke up earlier, and reported poorer sleep quality than at home.^20^ Unfortunately, no comparison studies investigated the effects of hospitalizations on sleep quality in lung transplant recipients.

In this study, symptoms of depression and anxiety were also significantly associated with sleep quality, as higher HADS depression and anxiety scores were noted among subjects with poor sleep. This is not surprising as a systematic review by Seiler et al^12^ noted that lung transplant recipients’ most common mental health conditions included major depression, panic disorders, and generalized anxiety disorders. Although the literature surrounding mental health outcomes after lung transplantation is dated, rates of these disorders are substantially higher than those of the general population and may even exceed rates in individuals with other chronic diseases.^21^ According to the systematic review by Seiler et al,^12^ major depression occurred in 26%**-**30% of lung transplant recipients within the first-year posttransplant. Depressed lung transplant recipients are at a higher risk for increased mortality due to reduced medication adherence, diminished coping, heightened likelihood of frailty, and decreased attendance to pulmonary rehabilitation, resulting in increased hospitalizations, graft failure, and infections.^21–24^

The findings from this study demonstrated that higher anxiety scores on the HADS anxiety subscale were significantly associated with a 1.34 greater likelihood of reports of poor sleep quality. Contrary to depression, anxiety was not associated with increased mortality following lung transplantation;,^21^ but only a few studies established the relationship between anxiety and sleep quality^17,22^ and none investigated these concepts in the context of hospitalization. A systematic review by Cordoza et al^22^ noted that the most common factors associated with poor sleep quality or insomnia were anxiety and depression in transplant candidates and recipients, concordant with this study.

Surprisingly, with several physiological factors explored for their potential to impact sleep, no significant relationships were found with poor sleep quality. This was discordant with other literature, which reflected associations with poor sleep in those lung transplant patients who were younger^16,18,25^ and were females.^16,25^ This divergence could be because a different instrument was used to measure sleep quality, namely the Insomnia Severity Scale, in studies by Yo et al^25^ and Rohde et al^26^ The Insomnia Severity Scale is an instrument that evaluates insomnia with attention to the severity of sleep onset, sleep maintenance, early morning awakenings, satisfaction with current sleep pattern, interference with daily functioning, and noticeability of sleep problems by others.^27^ The heterogeneous measures may account for these variable results, given the lack of agreed-upon conceptual and operational definitions of sleep quality as a construct. However, the main factor contributing to poor sleep quality in this research was psychological.

The study has several limitations. First, this study involved recipients from a single transplant center. However, the demographic characteristics and survival statistics observed in the sample align with national trends, supporting the relevance of the findings beyond the center where data were collected. Future research will benefit from multicenter studies with larger cohorts. Secondly, lung transplant recipients’ sleep quality was examined only at one point, which could have been different for individual recipients and at various times. Sleep is dynamic and may vary for individuals at various stages after transplant; hence, longitudinal studies would enhance understanding of how sleep quality changes over time in this population of patients. Thirdly, there is a possibility that the significant predictors of poor sleep in the sample were affected by factors omitted in this study. As an example, the survey did not gather data on the pretransplant pulmonary diagnoses leading to the need for lung transplantation. Although sleep quality is not regularly assessed in individuals with underlying Chronic Obstructive Pulmonary Disease or Interstitial Lung Disease,^28,29^ this could potentially be an important variable influencing sleep outcomes post-transplant. Also, participants were asked to self-report if they had been diagnosed with a sleep disorder like obstructive sleep apnea. With 21% of subjects self-reporting such a diagnosis, it remains uncertain whether sleep disorders were present before the transplant, developed after the transplant, or other sleep disorders might have been overlooked implicating a relationship with sleep quality. This is particularly important since studies on sleep-disordered breathing after lung transplantation are scarce. However, the largest prospective study that included 219 lung transplant recipients, found that 57.5% of these patients had sleep-disordered breathing.^30^

Other potential associations not well-explored included immunosuppression-related sleep disturbances, especially the dosing effects of Tacrolimus and corticosteroids. Rohde et al^26^ found that insomnia was more common in patients with higher exposure to Tacrolimus due to its influence on sleep cycles. Corticosteroids can also negatively affect sleep by altering the hypothalamic-pituitary-adrenal axis, disrupting the sleep-wake cycle, and causing hyperarousal.^31^ And although there was a positive association between poor sleep quality and treatment of acute rejection, it is unclear whether the diagnosis of rejection itself may trigger psychological and physiological changes affecting sleep; this could be due to excessive worry or anxiety over the possible loss of the graft and side effects caused by the pharmacotherapy used to treat rejection (ie, corticosteroids).

Finally, it is important to account for the possibility of self-report bias given the nature of the study. For example, due to social desirability bias, there was a possibility that individuals might underestimate their height and weight measures necessary for the calculation of the BMI. Similarly, recall bias might influence the accuracy of recalling events from the past 30 days, leading to either an overestimation or underestimation of the sleep quality calculation with PSQI.

Despite these limitations, several unique aspects of the study contribute to the body of knowledge pertaining to sleep in the context of lung transplantation. Future research recommendations include continued examination of predictors of poor sleep. Relationships between certain factors and sleep quality may have a bi-directional relationship where they can exacerbate or contribute to each other.^22^ For example, examining whether sleep quality influences its predictors and the extent to which it does may illuminate additional information about the interplay among these different variables. This would be most valuable with the employment of longitudinal designs to determine the bi-directional relationships of factors on sleep and at various time points after lung transplant. Given the complicated scoring system involved with the PSQI, other patient-reported outcome measures need to be developed to include ease of administration and scoring in an office or hospital setting.

Future studies using mixed methods methodology can play a role in a more holistic understanding of poor sleep after transplant. When examining the experience of sleep quality, mixed methods methodology may provide an additional dimension transcending reductionism and leading to the acquisition of knowledge of the irreducible wholeness of a lived experience. Finally, further research is needed to focus on the development of interventions to address mental health and sleep quality issues prominent in lung transplant recipients.

## Conclusions

Poor sleep was very prevalent after lung transplantation. Recipients with anxiety symptoms were at a higher risk for poor sleep. A better understanding of the relationships between poor sleep and sleep quality factors requires further research to guide the development of patient-centered screening and practice interventions to identify those at risk for sleep problems.
